# Chemisty Via Dentistry

**Published:** 1883-08

**Authors:** N. L. S. Buckner


### Chemistry via. Dentistry.

BY DR. N. L. S. BUCKNER.

[Read Before the Kentucky State Dental Society.]

The object of this paper is to so introduce the subject of chemistry, as to create a general discussion and exchange of ideas of those present, rather than to give any elaborate details of the subject. The desire now for chemical knowledge is on the increase, and step by step we are advancing to higher professional attainment in this as in other departments of our work. Already our attainment in this field is large, but how much larger the space covered by our ignorance than by our knowledge. A work so well begun ought to be assiduously prosecuted.

Let the magnetic influence possess our thought, so that by studying, reflecting and learning, we shall very soon develop our profession in this science to the very highest standard. The science of chemistry has for its object the study of the nature and properties of all the materials which enter into the composition or structure of the earth, the sea, and the air, and of the various organized or living beings which inhabit them.

In a special department of this subject, we are made to feel the necessity of a careful selection of our course, through the abundant material unfolded in us, above us, below us and around us, in such wondrous harmony. To consider the natural organs of mastication and to examine the chemical principals upon which the success or failure of the artificial substances used by dentists to remedy defects in those organs will necessiate the study of physical chemistry. This, of course, includes a knowledge of chemical composition of the teeth, as well as of the soft parts which surrounds them, and the secretions which flow over them. No other portion of the human organism is of such complex structure as the mouth, and no other has such diversified duties to perform. It is needless here to give an outline of that wonderfully constructed laboratory, where each chemical element joins with different fluids to produce (in health) that greatest and best of all created things, a bright, healthy man.

THE SECRETIONS.

It is only necessary to mention that the secretions of the mouth in its normal condition is slightly alkaline and acid. The parotid, submaxilary, and the sublingnal glans vary the condition of this fluid, as well as that produced from the numerous glans of the lips, cheek, gums, tongue, etc.

These different and destructive secretions all unite to form the fluid ordinarily termed salvia.

By this mixed fluid the mouth is constantly moistened, assisting speech, desolving soluable substances for the enjoyment of taste, lubricating the surfaces of the teeth, favoring the transmission to the stomach, and assisting in the subsequent solution of the latter by the fluids of the stomach. It has also a certain chemical effect upon the food which is the first in the series of chemical process (digestion) by which the food is prepared for the use of the economy of nature. Besides this, we have another effect which marks its destruction chiefly upon the teeth, commonly known as *decay*.

DECAY.

It is generally understood that the existing causes of decay are chiefly different forms of chemical actions, which may either follow the use of acids as food, or medicine, or may result from a vitiation of the secretions of the mouth, either from a systemic derangement, or from local cause, indicating acid saliva, or from fermentation and decomposition of animal or vegitable substances lodged about the teeth.

TEETH.

The texture of the teeth has much to do in resisting this active agent. An acid will act with greater energy on a soft, porous tooth, than on one of firmer structure; yet the chemical result is the same. There is difference of opinion as to the particular acid producing the several kinds of decay.

ACIDS.

*Nitric-acid* is the supposed prominent agent in the production of *white decay*, *sulphuric-acid* of *black decay* and *hydrochloric-acid*, of the *brown* variety.

We find also a fourth-class, commonly called *chemical abrasion*, in which the entire tooth substance is removed, as far as the active agent has extended its work.

### SALIVARY CALCULUS.

In certain conditions of the saliva, we have still another form of chemical element, precipitated from the saliva and lodged upon the teeth, called *salivary calculus*. Its composition is almost entirely of earthy or of inorganic ingredient, and its action upon the gums and teeth very destructive.

### GALVANIC DISTURBANCE

Have also serious chemical trouble when two, unlike metal fillings, touch, and a favorible condition of the oral fluids is maintained; a galvanic disturbance is permitted; if not in actual contact, a shock may be produced when the metals are connected by the tongue, cheek, or saliva. This trouble usually occurs during mastication, and varies in intensity.

### EXAMINATION.

Every dentist, after thorough examination of the mouth, ought to be able, from the appearance, and from accompanying circumstances, to tell exactly what chemical agent induced the trouble. He should know, also, the composition, the source or origin, and the *modus operandi* of the agent, as well as its antidote, and the proper mode of preventing its formation.

### ALLOYS, ETC.

The study of the nature and prospects of the alloys is an interesting one, as they are not only mixtures of metals possessing certain distinct qualities, but in reality are true chemical compounds.

### NATURAL PHYLOSOPHY.

This division, however, embraces the changes by heat, electricity, magnetism, and the attractive forces, whose laws and effects lie within the province of natural philosophy, and not in the scope assigned this paper.

CONCLUSION.

Since rapidly scanning this subject, it will be seen that it might have been indefinitely elaborated; but I prefer to indicate what seems to be its bearings, and leave the intelligent to draw their conclusions. This attempt has not been to introduce a hypothesis of chemical arrangement, or constitute a code for the attainment of chemical analysis, but to call attention to chemical phenomena, and trace its destruction upon the living and dead structures of the oral cavity.

